# PROTOCOL: Psychometric properties of instruments for measuring elder abuse and neglect in community and institutional settings: A systematic review

**DOI:** 10.1002/cl2.1342

**Published:** 2023-06-27

**Authors:** Fadzilah Hanum Mohd Mydin, Christopher Mikton, Wan Yuen Choo, Ranita Hisham Shanmugam, Aja Murray, Yongjie Yon, Raudah Mohd Yunus, Noran Naqiah Hairi, Farizah Mohd Hairi, Marie Beaulieu, Amanda Phelan

**Affiliations:** ^1^ Department of Primary Care Medicine, Faculty of Medicine Universiti Malaya Kuala Lumpur Malaysia; ^2^ Department of Social Determinants of Health World Health Organization Geneva Switzerland; ^3^ Department of Social and Preventive Medicine, Faculty of Medicine Universiti Malaya Kuala Lumpur Malaysia; ^4^ Universiti Malaya Library Universiti Malaya Kuala Lumpur Malaysia; ^5^ Department of Psychology University of Edinburgh Edinburgh United Kingdom; ^6^ World Health Organization Regional Office for Europe Copenhagen Denmark; ^7^ Department of Public Health Medicine Universiti Teknologi MARA Sungai Buloh Malaysia; ^8^ Faculté des lettres et sciences humaines, École de travail social, Centre de recherche sur le vieillissement, CIUSSS Estrie-CHUS Université de Sherbrooke Québec Canada; ^9^ School of Nursing and Midwifery Trinity College Dublin Dublin Ireland

## Abstract

**Background:**

The psychometric properties of elder abuse measurement instruments have not been well‐studied. Poor psychometric properties of elder abuse measurement instruments may contribute to the inconsistency of elder abuse prevalence estimates and uncertainty about the magnitude of the problem at the national, regional, and global levels.

**Objectives:**

The present review will utilise the COSMIN taxonomy on the quality of outcome measures to identify and review the instruments used in measuring elder abuse, assess the instrument's measurement properties, and identify the definitions of elder abuse and abuse subtypes measured by the instrument.

**Search Methods:**

Searches will be conducted in the following online databases: Ageline, ASSIA, CINAHL, CNKI, EMBASE, Google Scholar, LILACS, Proquest Dissertation & Theses Global, PsycINFO, PubMed, SciELO, Scopus, Sociological Abstract and WHO Index Medicus. Relevant studies will also be identified by searching the grey literature from several resources such as OpenAIRE, BASE, OISter and Age Concern NZPotential studies by searching the references of related reviews. We will contact experts who have conducted similar work or are currently conducting ongoing studies. Enquiries will also be sent to the relevant authors if any important data is missing, incomplete or unclear.

**Selection Criteria:**

All quantitative, qualitative (that address face and content validity), and mixed‐method empirical studies published in peer‐reviewed journals or the grey literature will be included in this review. Studies will be included if they are primary studies that (1) evaluate one or more psychometric properties; (2) contain information on instrument development, or (3) perform content validity of the instruments designed to measure elder abuse in the community or institutional settings. Studies should describe at least one of the psychometric properties, such as reliability, validity and responsiveness. Study participants represent the population of interest, including males and females aged 60 or older in community or institutional settings (i.e., nursing homes, long‐term care facilities, assisted living, residential care institutions, and residential facilities).

**Data Collection and Analysis:**

Screening of titles, abstracts, and full texts of the selected studies will be evaluated based on the preset inclusion criteria by two reviewers. Two reviewers will be assessing the quality appraisal of each study using the COSMIN Risk of Bias checklist and the overall quality of evidence of each psychometric property of the instrument against the updated criteria of good measurement properties. Any dispute between the two reviewers will be resolved through discussions or consensus with a third reviewer. The overall quality of the measurement instrument will be graded using a modified GRADE approach. Data extraction will be performed using the data extraction forms adapted from the COSMIN Guideline for Systematic Reviews of Outcome Measurement Instruments. The information includes the characteristic of included instruments (name, adaptation, language used, translation and country of origin), characteristics of the tested population, psychometric properties listed in the COSMIN criteria, including details on the instrument development, content validity, structural validity, internal consistency, cross‐cultural validity/measurement invariance, reliability, measurement error, criterion validity, hypotheses testing for construct validity, responsiveness and interoperability. We will perform a meta‐analysis to pool psychometric properties parameters (where possible) or summarise qualitatively.

## 1 BACKGROUND

### 1.1 The problem, condition or issue

Elder abuse is now recognised as a prevalent and growing issue with profound concerns for older people's health and social well‐being. Elder abuse is ‘a single or repeated act, or lack of appropriate action, occurring within any relationship with an expectation of trust, which causes harm or distress to an older person's (World, 2022). This type of violence violates human rights and includes physical, sexual, psychological and emotional abuse; financial and material abuse; abandonment; neglect; and serious loss of dignity and respect. It also involves deliberate or neglectful acts by the older person's formal or informal caregiver or trusted individual that cause harm to a vulnerable older person. Elder abuse can occur in various settings, including at home, in institutional settings and the community. The definition of different subtypes of elder abuse based on the Centers for Disease Control and Prevention are provided in Supporting Information: Appendix 1 (Hall, 2016).

Elder abuse is a complex issue that can have serious consequences for older adults. Several risk factors can increase the likelihood of elder abuse occurring among older adults, including social isolation, cognitive impairment, physical dependence, mental health issues, substance abuse, history of abuse, financial exploitation, low income and socioeconomic status, women gender, racial/ethnic groups and financial dependence (Johannesen, 2013; Li, 2020; Pillemer, 2016; Storey, 2020; Yan, 2015). Perpetrator risk factors include poor psychological health, substance misuse, and abuser dependency. Older adult and perpetrator relationships and marital status are potential elder abuse risk factors. There are differences between elder abuse in the community and institutional settings regarding types of abuse, perpetrators and reporting procedures. Abuse of elders in the home or community is mainly perpetrated by their spouses, family members or caregivers responsible for caring for the older adults, while professional caregivers or peers commit abuse in the institutional setting (Yon, 2019; Yon, 2019a). Abuse in the community and institutional settings can take various forms, such as physical, emotional, financial, sexual and neglect. However, other types of abuse may also exist in the institutional setting, such as medication errors, over‐medication or restraint use. Reporting elder abuse in the community can be challenging because the older adult may be isolated or afraid to report the abuse. In institutions, staff members are mandated to report suspected abuse to the appropriate authorities.

Given the rapidly ageing demographic changes, the issue of elder abuse urgently requires the attention and intervention of healthcare providers, social welfare agencies, and policymakers. Data from adult protective agencies suggests that elder abuse is often underreported in many countries. The prevalence of elder abuse among older people is established through population surveys. Older adults or their proxies are surveyed directly to collect information about their experience, including exposure frequency and specific types of abuse. A recent systematic review estimated that the prevalence of elder abuse in the community was 15.7% over the past 1‐year based on population surveys (Yon, 2017). In institutional settings such as nursing homes and other long‐term care facilities, there was insufficient data to estimate the prevalence reported by older adults, but findings found that approximately two in three staff members working in nursing homes admitted to perpetrating elder abuse in the past year (Yon, 2017; Yon, 2019a). The major drawbacks to the quantified estimates in these reviews were that the data lacked comparability due to the heterogeneous methods; most of the included studies are from high‐income countries. Hence, there is a lack of robust prevalence studies in low‐ and middle‐income countries. However, over the past 10 years, research on elder abuse are emerging from developing countries such as Malaysia, Iran, Brazil and India in which performing a new review of the elder abuse measurement instruments is needed (Blay, 2017; Nassiri, 2016; Patel, 2018; Sooryanarayana, 2013). The research findings from countries worldwide may lead to identifying similarities and differences in elder abuse measurement instruments. A comprehensive review of the psychometric properties of the measurement instruments utilised in the prevalence studies can help to identify the gaps and provide relevant recommendations for elder abuse measurement instruments.

It is important to assess the burden of elder abuse in a population, comparing the prevalence of elder abuse in different populations to identify risk factors and examine trends to plan and evaluate strategies, policies or large‐scale interventions. However, the literature has demonstrated a wide variation in reported prevalence rates due to the methodological differences and lack of agreement on defining and measuring elder abuse and its subtypes (Sooryanarayana, 2013; World, 2022; Yan, 2015; Yon, 2017; Yon, 2019; Yon, 2019a; Zhang, 2022). This issue is not limited to elder abuse but is a common problem researchers face measuring exposure to other types of violence, such as child maltreatment and interpersonal violence (Alhabib, 2010; Mathews, 2020). In addition, reports have also documented extensive cultural variation in the circumstances and context of elder abuse (Lee, 2014; Li, 2020; Zhang, 2019). Most prevalence studies applied the widely accepted definition of elder abuse and its subtypes adopted by the World Health Organisation and the United States Centres for Disease Control and Prevention (Hall, 2016; Sooryanarayana, 2013; Yon, 2017; Yon, 2019a). Besides this overarching framework, there are various words used interchangeably to describe the phenomenon, such as ‘harm’, ‘exploitation’, ‘mistreatment’, ‘maltreatment’, and ‘violence’ found in the literature. To what extent researchers adopted these terms or whether standardised (or non‐standardised) instruments were used to measure ‘abuse’ in these studies remains unclear. Although there is no gold standard measurement instrument currently to establish the prevalence of elder abuse in the community or institutional settings, many elder abuse measures emerged for this purpose over the past decades.

Despite the availability of several elder abuse measurement instruments, most of the studies only document part of the psychometric properties of these instruments (Cooper, 2008; Jackson, 2018; Sooryanarayana, 2013; Yan, 2015; Yon, 2017; Yon, 2019a; Zhang, 2022). Besides, adapting an existing elder abuse instrument to be used in another population is a common practice, yet information regarding the cross‐cultural validity of the instrument remains sparse. Information regarding the adaptation process is important when an instrument is used in a different gender, community, language, setting and time to avoid introducing bias into a study. Measurement instruments must also be attuned for cultural suitability, in which new measurement instruments should have cognitive interviews with the relevant population and cross‐cultural validity being assessed (Prinsen, 2018; Terwee, 2018).

Prevalence studies conducted using poor or unknown‐quality measurement instruments may produce inaccurate findings. Extensive evaluation of the quality of these instruments would provide evidence of how their measurement properties were assessed and could help researchers in this field when choosing a measurement tool that is valid and reliable in the future.

### 1.2 Description of the phenomena of interest

The psychometric properties of elder abuse measurement instruments used in existing elder abuse prevalence surveys have not received enough attention (Cooper, 2008; Sooryanarayana, 2013; Yan, 2015; Yon, 2017; Yon, 2019a; Zhang, 2022). Selecting the best measurement instruments for elder abuse prevalence studies requires an instrument supported by evidence of reliability, validity and responsiveness (Prinsen, 2018; Terwee, 2018). Evaluating the psychometric properties of the existing measurement instruments can identify the gaps in the knowledge of the psychometric evidence and guide in designing new development of elder abuse measurement instruments’ psychometric properties.

This systematic review will utilise the Consensus‐based Standards for selecting health Measurement Instruments (COSMIN) methodology to conduct a systematic review of the psychometric properties of elder abuse measurement instruments (Prinsen, 2018). COSMIN methodology provides a comprehensive checklist to assess the quality and criteria for the good measurement properties of the instruments utilised for research and practice. The COSMIN taxonomy of psychometric properties is based on three domains: reliability, validity and responsiveness. The first domain, the reliability of the measurement instrument scores, comprises internal consistency, reliability (test‐retest, inter‐rater and intra‐rater) and measurement error (test‐retest, inter‐rater and intra‐rater). The second domain, the validity of the measurement instrument, includes content validity (relevance, comprehensiveness or comprehensibility including face validity), structural validity, hypotheses testing for construct validity, and cross‐cultural and criterion validity. The third domain is the responsiveness of measurement instruments, defined by the single property of responsiveness to intervention. The definition of each domain is available in the COSMIN manual for systematic reviews of Patient Reported Outcome Measures (Prinsen, 2018).

### 1.3 Why it is important to do this review

Elder abuse is also a serious public health and social epidemic expected to escalate (Pillemer, 2016). The World Health Organization Global Action Plan on Ageing and Health Strategy and the United Nations Decade of Healthy Aging (2021–2030) outline the need to establish the prevalence of elder abuse and implement evidence‐informed elder abuse prevention and response programs (United Nations, 2015). Similarly, the 2030 Agenda for Sustainable Developmental Goal (SDG) acknowledges human rights within all categories of age in society, concentrating on vulnerable groups, including older people, which also aims to end discrimination later in life (Lee, 2016). Valid data is needed for older people in line with the SDG indicator (SDG 16.1.3) that focuses specifically on the proportion of the population in each country subjected to different subtypes of abuse, including physical and psychological violence and sexual abuse across all ages, including older people.

Based on an initial search, there is a systematic review of survey instruments used to measure staff‐to‐resident elder abuse in residential care settings (Malmedal, 2020). However, this review excluded the studies on elder abuse in the community. There is also a narrative review on instruments to measure violence against older women (unpublished) (Mikton, 2019). A review by Jackson (2018) focused on financial exploitation among older people (Jackson, 2018), while several other reviews have focused on elder abuse screening and detection instruments used by service providers in healthcare settings and home environments (Gallione, 2017; McCarthy, 2017; Van, 2020). Though these screening and detection instruments are critical for service providers to detect and respond to potential cases of abuse, they may not help assess the prevalence of elder abuse.

There are gaps in the literature on elder abuse regarding the real magnitude of the problem worldwide, which may mask the challenges older people face in implementing policy and preventive programmes. There is a lack of a systematic review that conducts an in‐depth analysis of the measurement instruments by examining the construct definition and psychometric properties used to measure the prevalence of elder abuse. There is no systematic review of the psychometric properties of the elder abuse measurement instruments and the underlying definitions of elder abuse (Gallione, 2017; Malmedal, 2020).

A systematic review of the psychometric properties of elder abuse measurement instruments conducted using the ten steps‐procedure as recommended in the Consensus‐based Standards for the Selection of health measurement instruments (COSMIN) methodology is needed to examine the definitions and items used to measure the prevalence of elder abuse in the community or institution settings (Mokkink, 2018; Prinsen, 2018; Terwee, 2018). This approach will advance our understanding of the construct definition of elder abuse and its measurement used in practice, research and policy worldwide.

A comprehensive and high‐quality systematic review can contribute to evidence‐based recommendations for the appropriate elder abuse measurement instrument worldwide. The findings of this review will contribute to identifying or developing a standardised, accurate and valid approach to measuring elder abuse in the community or institutional settings. This will enable practitioners and policymakers to make an informed choice when selecting an instrument to measure elder abuse and employ an evidence‐informed approach for assessing elder abuse initiatives in the future. There is also the possibility that no pre‐existing measure is currently up to the task, thus motivating the need for a new one. This work will inform the development of new measure(s) in the (likely) event. If the review finds that there is a good instrument that can either be used as is or will require some further refinement and cross‐cultural testing, and that will be done with the authors of the instrument; or the review finds there are no decent instruments and the new instrument is developed, but drawing on existing instruments, sub‐scales, and items as much as possible.

## 2 OBJECTIVES

Our research questions are:

1. What are the psychometric properties of elder abuse measurement instruments in the community or institutional setting?
2. What is the quality of the psychometric properties of elder abuse measurement instruments?
3. What is the definition of elder abuse used in the elder abuse measurement instruments?
4. What are the subtypes of abuse measured in the elder abuse measurement instruments?

We aim to gather, critically appraise, compare and describe all current instruments used to measure elder abuse in the community or institutional settings and their psychometric properties. We will also identify the definitions and domains of elder abuse used in these measures. Based on our findings, we will identify all instruments used internationally in prevalence studies of elder abuse and determine their comprehensiveness in measuring elder abuse. Where possible, we will recommend the most promising instrument (s) on which to draw to develop a reliable, valid (including cross‐culturally valid), and responsive instrument to be used in such studies. Where no suitable instruments are identified, we will provide recommendations for future research to improve elder abuse measurement.

## 3 METHODS

### 3.1 Criteria for considering studies for this review

#### 3.1.1 Types of studies

The eligible studies should at least aim (1) to evaluate one psychometric property; (2) contain information on instrument development; or (3) perform the content validity of the elder abuse measurement instruments in the community or institutional settings. We will also include studies in which elder abuse measurement instruments are used in a validation study of another instrument.

We will exclude studies that used elder abuse measurement instruments solely for screening purposes to establish a diagnosis in clinical or hospital settings (such as emergency departments) or cases from adult protective services. However, we will include instruments used for screening if they are utilised in prevalence studies and at least describe the instrument development, perform content validity or evaluate a minimum of one psychometric property.

#### 3.1.2 Types of participants

The study sample should represent the population of interest, including males and females aged 60 or older in community or institutional settings(i.e., nursing homes, long‐term care facilities, assisted living facilities, residential care institutions, residential facilities, and skilled nursing facilities), in all countries of the world.

#### 3.1.3 Phenomena of interest

Instrument development, content validity and psychometric properties of elder abuse measurement instruments in the community or institutional settings.

#### 3.1.4 Types of outcome measures

The psychometric properties outlined by the COSMIN Taxonomy for quality of measurement outcome domains will be adhered to (Prinsen, 2018). These include three quality domains: reliability, validity, and responsiveness.

We will also list all available elder abuse measurement instruments, which at least reported instrument development, content validity or evaluate one or more psychometric properties, elder abuse definition, and the abuse subtypes used in each instrument.

##### Primary outcomes

The psychometric properties of the instruments for elder abuse prevalence measurement in the community or institutional settings will be reported. The psychometric properties listed in the COSMIN criteria include the following:

1. Instrument development
2. Content validity
3. Structural validity
4. Internal consistency
5. Cross‐cultural validity\measurement invariance
6. Reliability
7. Measurement error
8. Criterion validity
9. Hypotheses testing for construct validity
10. Responsiveness.

The definition of each psychometric property in Supporting Information: Appendix 2 is similar to the description provided in the COSMIN methodology for systematic reviews of the Patient‐Reported Outcome Measures (PROMs) manual (Mokkink, 2018; Prinsen, 2018; Terwee, 2018).

##### Secondary outcomes

Other outcomes to be reported include the definition of elder abuse and its subtypes used in the measurement instruments.

#### 3.1.5 Types of settings

We will include all studies conducted in the community or institutional settings (i.e., nursing homes, long‐term care facilities, assisted living facilities, residential care institutions, residential facilities, and skilled nursing facilities) worldwide.

### 3.2 Search methods for identification of studies

An information specialist (RH) will design a primary search strategy that consists of a combination of search terms using the medical subject heading (MeSH) and free text terms that consist of ‘elder abuse’ ‘elder mistreatment’, ‘elder maltreatment’, ‘elder neglect’ AND ‘psychometric’ OR ‘outcome assessment’ OR reproducible OR reliability OR validity OR ‘screening tool’ OR ‘screening assessment’ OR assessment OR ‘assessment tool’ OR screening OR ‘appraisal tool’. The search strategy will be developed, revised by content experts, and piloted in several rounds to improve its sensitivity and specificity. The final strategy will be completed in PubMed and replicated in other databases. The final search strategy is available in Supporting Information: Appendix 3.

Our sources of information will include electronic databases, trial registries, and grey literature. An electronic search will be performed searching the title, abstract, and keywords through AgeLine via EBSCOhost, ASSIA via ProQuest, CINAHL via EBSCOhost, EMBASE, LILACS, Proquest Dissertation & Theses Global, PsycINFO via EBSCOhost, PubMed, SciELO, Scopus, Sociological Abstract via ProQuest, Chinese National Knowledge Infrastructure (CNKI), Korean Citation Index (KCI), and WHO Index Medicus.

We will consider only articles that are published or in the press. We will not limit the date of acceptance or publication.

#### 3.2.1 Searching other resources

Searching other resources: Relevant studies will also be identified by searching the grey literature from several resources, such as Google Scholar (first 200 results), OpenAIRE, BASE, OISter, and Age Concern NZ. Direct searches of the entire journal content will be done in prominent academic journals that publish empirical elder abuse research, such as the Journal of Elder Abuse and Neglect, Journal of Interpersonal Violence, Journal of Adult Protection, and Journal of Trauma, Abuse, and Violence.

Citations and reference list: Potential studies that may not have been identified through the searches will be identified by searching the references of related articles or reviews. Forward citation searches will be used to identify cited studies included in these related articles or reviews.

Contacting experts: We will contact experts who have conducted similar work or are currently conducting ongoing studies.

### 3.3 Data collection and analysis

#### 3.3.1 Description of methods used in primary research

Quantitative, qualitative (addressing face and content validity) and mixed‐method empirical studies.

#### 3.3.2 Selection of studies

We will use EndNote and EPPI Reviewer web applications to manage all documents retrieved throughout the search process. Before the screening, all duplicates will be removed in EndNote. All citations will be imported into the EPPI Reviewer web application (Thomas, 2010). We will upload the full text for screening, data extraction, and analysis in the EPPI‐Reviewer web application.

Two reviewers will independently perform the primary screening for studies based on the titles and abstracts. The selected articles will then be grouped into relevant, irrelevant and unsure. Articles deemed irrelevant by both reviewers will be excluded from the review. A list of to‐be‐included articles will be prepared. If there is no consensus between the two reviewers, the third or whole team will discuss it to make the final decision.

Next, the full text of selected studies will be obtained and uploaded to the EPPI Reviewer web application. Two reviewers will independently review the full text to determine their eligibility for inclusion. The studies that do not meet the inclusion criteria will be excluded from the review, and reasons for exclusion will be provided. Any disagreements will be resolved through discussion with a third reviewer. Multiple publications or reports of the same instrument will be explored and reported.

The screening and selection process will be reported in the final report and outlined in a PRISMA flow diagram (Moher, 2009).

#### 3.3.3 Data extraction and management

Two reviewers will independently extract the data from the selected full text and enter it into the data extraction form to reduce bias and errors. Articles other than the English language will be translated into English using Google Translate or an expert in the language. A third reviewer will check for any differences. Reviewers will discuss any potential difference with the team, and if unresolved, the manuscript authors will be contacted for additional information. Enquiries will also be sent to the relevant authors to determine if any important data is missing, incomplete or unclear. The information will be recorded as missing if there are no responses after two reminders.

Information and the details of the interpretability and feasibility aspects of the measuring instruments will be extracted and described for each measurement instrument, which will be utilised to assess if the results of different studies can be pooled or summarised qualitatively (Prinsen, 2018).

Data on the characteristic of the included measuring instruments, such as the name of the measurement instruments, constructs being measured, target population, original language, available translations in other languages, results of the psychometric properties such as number of scales or subscales, number of items per subscale, response options, and recall period as shown in Table 1 (Supporting Information: Appendix 4). The following information will also be extracted; study population, types and subtypes of abuse measured, the definitions used, and the details of instruments administration such as the country, language and response rate as shown in Table 2 (Supporting Information: Appendix 5).

We will also extract the information based on the psychometric properties listed in the COSMIN criteria, including information on the instrument development, content validity, structural validity, internal consistency, cross‐cultural validity/measurement invariance, reliability, measurement error, criterion validity, hypotheses testing for construct validity and responsiveness.

Information on the definition of elder abuse used and subtypes measured in the measurement instruments will also be extracted as the secondary outcome of this review. We will also map the definition used in each measurement instrument.

#### 3.3.4 Methodological and quality assessment of included studies

All assessments will be performed based on recommendations proposed in the COSMIN guideline (Mokkink, 2018). Four steps are involved in this process. First, we evaluate the methodological quality of the instrument development of included studies based on the COSMIN Risk of Bias checklist (Mokkink, 2018; Prinsen, 2018). Second, we will evaluate every study's measurement property against the updated criteria for good measurement properties as sufficient (+), insufficient (−) or indeterminate (?). Third, we will summarise each measurement property from each instrument and then apply the modified grading of Recommendations Assessment, Development and Evaluation (GRADE) to determine the quality of the overall evidence for each instrument. Finally, we will identify the application and suitability of these instruments based on evidence of sufficient measurement properties.

1. Assessment of risk of bias
  Critical appraisal of articles will be performed using the COSMIN Risk of Bias checklist to assess the methodological quality of each study based on the proposed measurement properties to be assessed (Mokkink 2018). There are ten boxes in the COSMIN Risk of Bias checklist, as shown in Table 3 (Supporting Information: Appendix 6).
  Each study will be rated using a four‐point rating system where each standard within a COSMIN criteria box will be marked as ‘4 = very good, ‘3 = adequate’, ‘2 = doubtful’, or ‘1 = inadequate’. If multiple studies on different aspects of measurement properties of the same instrument are published, each study will be evaluated separately, considering each has specific design requirements. The COSMIN Risk of Bias checklist can be used as a modular tool to assess each study by completing the boxes relevant to the psychometric properties assessed in each article. An overall judgement will be made on the methodological quality of each study. The overall rating of the individual study will be based on the lowest rating of any standard (i.e., the worst score counts principle) on the checklist.
2. Evaluation of measurement properties based on updated criteria for good measurement properties
  Two independent reviewers will assess all study results for each included study and measure according to the COSMIN guidance on updated criteria of good measurement properties (Prinsen, 2018).
  Content validity will be evaluated based on the content validity of the measure itself and the quality of the available studies (Terwee et al., 2018). Content validity will be scored as sufficient (+), insufficient (−), indeterminate (?), and inconsistent (±) based on existing development studies, content validity studies, and reviewer ratings.
  The results of other psychometric properties (reliability, validity and responsiveness) of each study will be evaluated according to the updated criteria for good psychometric properties either as sufficient (above the quality criteria threshold: ‘+’), insufficient (below the quality criteria threshold: ‘−’) or indeterminate (less robust data that do not meet the quality criteria: ‘?’).
  Any discrepancies will be solved by consensus among the two reviewers or with the help of a third reviewer.
  We will present the evidence summary, including the reviewed measurement instruments, the outline or pooled result of each psychometric property, and the overall rating and quality of the evidence for each instrument.
3. Summarise the quality of PROMS and grading the evidence
  All analyses will be performed based on recommendations proposed in the COSMIN guideline. We will compare the overall results based on the criteria to determine if the measurement property for the instrument is sufficient (+) or insufficient (−), or inconsistent (±).
  To conclude the quality of the instruments, the results of all available studies for each measurement property are assessed for consistency. For consistent results, all studies will be summarised qualitatively or pooled quantitatively by performing a meta‐analysis, provided there is adequate quantitative data on psychometric properties. For example, intraclass correlations from different studies assessing the same PROM will be pooled by calculating the weighted means and its 95% confidence interval based on the sample size in each study.
  For inconsistent results, we will further explore the reasons behind this inconsistency, including examining the populations, methods used, or quality of the studies. We will then summarise the consistent results based on these subpopulations. If there is no explanation for the inconsistent findings, the overall rating will be inconsistent (±). For example, the overall rating for a particular measurement property, such as reliability, may be sufficient (+) for measuring abuse among community‐based older persons but insufficient (−) in institutionalised older people. However, if the inconsistent results cannot be explained. In that case, the overall rating will be based on the majority outcome of the results rated as sufficient if at least 75% of the studies have consistent results; otherwise, we will downgrade the evidence to insufficient (Prinsen, 2018).
  The quality of the evidence will be graded using the modified GRADE approach into four levels of evidence: high, moderate, low, and very low (Holger, 2013). The quality of evidence of the content validity is graded based on three factors: risk of bias, inconsistency and indirectness. Other psychometric properties are evaluated based on four factors: risk of bias, inconsistency, imprecision, and indirectness. Both the overall rating and quality of evidence will be reported.
  Two reviewers will summarise and assess the certainty of the overall evidence, and consensus among the two reviewers will be reached. A third reviewer or the rest of the team members will be consulted if there are any discrepancies.
4. Recommendations for instrument based on overall evidence and reporting of review

Finally, each summary of the findings table (one table per measurement property)will be utilised to provide recommendations for selecting the most appropriate measurement instrument that fits a particular context.

The review will be reported following the PRISMA statement (Moher, 2009).

## CONTRIBUTIONS OF AUTHORS

- Content: Fadzilah Hanum Mohd Mydin, Choo Wan Yuen, Christopher Mikton, Noran Naqiah Hairi, Farizah Hairi, Raudah Mohd Yunus, Marie Beaulieu, Amanda Phelan
- Systematic review methods: Fadzilah Hanum Mohd Mydin, Choo Wan Yuen, Christopher Mikton, Yongjie Yon, Noran Naqiah Hairi, Farizah Hairi, Raudah Mohd Yunus
- Statistical analysis: Choo Wan Yuen, Aja Murray, Fadzilah Hanum Mohd Mydin
- Information retrieval: Ranita Hisham Shanmugam

## DECLARATIONS OF INTEREST

Choo Wan Yuen, Noran Naqiah Hairi, Christopher Mikton, Yongjie Yon, Amanda Phelan, and Raudah Mohd Yunus are involved in the design, conduct and publication of a study potentially eligible for the review.

Fadzilah Hanum Mohd Mydin, Marie Beaulieu, Aja Murray, Farizah Hairi and Ranita Hisham Shanmugam have no conflict of interest to declare.

## PRELIMINARY TIMEFRAME

The approximate date for submission of the systematic review: November 2023.

## SOURCES OF SUPPORT

### Internal sources

- Ministry of Higher Education Fundamental Research Grant Scheme, Malaysia

(FRGS/1/2022/SKK05/UM/01/1) awarded to the principal investigator, Choo Wan Yuen

### External sources

- New Source of support, Other

No sources of support were provided.

## Supporting information

Supporting information.
