# Correction to ‘PROTOCOL: Psychometric properties of instruments for measuring elder abuse and neglect in community and institutional settings: A systematic review’

**DOI:** 10.1002/cl2.1365

**Published:** 2023-10-09

Mohd Mydin, F. H., Mikton, C., Choo, W. Y., Shanmugam, R. H., Murray, A., Yon, Y., Mohd Mydin, R., Hairi, N. N., Mohd Hairi, F., Beaulieu, M., & Phelan, A. (2023). PROTOCOL: Psychometric properties of instruments for measuring elder abuse and neglect in community and institutional settings: A systematic review. *Campbell Systematic Reviews*, **19**, e1342. https://doi.org/10.1002/cl2.1342

The **Search Methods** in the **Abstract**, and content in section **3.2 Search methods for identification of studies** have been corrected. The corrected sections are shown below:

**Abstract**

**Search Methods:** Searches will be conducted in the following online databases: AgeLine via EBSCOhost, ASSIA via ProQuest, CINAHL via EBSCOhost, EMBASE, LILACS, Proquest Dissertation & Theses Global, PsycINFO via EBSCOhost, PubMed, SciELO, Scopus, Sociological Abstract via ProQuest, Chinese National Knowledge Infrastructure (CNKI) and WHO Global Index Medicus. Relevant studies will also be identified by searching the grey literature from several resources such as Google Scholar, Campbell Collaboration, OpenAIRE, and GRAFT. We will contact experts who have conducted similar work or are currently conducting ongoing studies. Inquiries will also be sent to the relevant authors if any important data is missing, incomplete or unclear.

**3.2 | Search methods for identification of studies**

An information specialist (RH) will design a primary search strategy that consists of a combination of search terms using the medical subject heading (MeSH) and free text terms that consist of ‘elder abuse’ ‘elder mistreatment’, ‘elder maltreatment’, ‘elder neglect’ AND ‘psychometric’ OR ‘outcome assessment’ OR reproducible OR reliability OR validity OR ‘screening tool’ OR ‘screening assessment’ OR assessment OR ‘assessment tool’ OR screening OR ‘appraisal tool’. The search strategy will be developed, revised by content experts, and piloted in several rounds to improve its sensitivity and specificity. The final strategy will be completed in PubMed and replicated in other databases. The final search strategy is available in Supporting Information: Appendix 3.

Our sources of information will include electronic databases, trial registries, and grey literature. An electronic search will be performed searching the title, abstract, and keywords through AgeLine via EBSCOhost, ASSIA via ProQuest, CINAHL via EBSCOhost, EMBASE, LILACS, Proquest Dissertation & Theses Global, PsycINFO via EBSCOhost, PubMed, SciELO, Scopus, Sociological Abstract via ProQuest, Chinese National Knowledge Infrastructure (CNKI), Korean Citation Index (KCI), and WHO Index Medicus.

We will consider only articles that are published or in the press. We will not limit the date of acceptance or publication.

Searching other resources: Relevant studies will also be identified by searching the grey literature from several resources, such as Google Scholar (first 200 results), Campbell Collaboration, OpenAIRE, and GRAFT. Direct searches of the entire journal content will be done in prominent academic journals that publish empirical elder abuse research, such as the Journal of Elder Abuse and Neglect, Journal of Interpersonal Violence, Journal of Adult Protection, and Journal of Trauma, Abuse, and Violence.

Citations and reference list: Potential studies that may not have been identified through the searches will be identified by searching the references of related articles or reviews. Forward citation searches will be used to identify cited studies included in these related articles or reviews.

Contacting experts: We will contact experts who have conducted similar work or are currently conducting ongoing studies.

We apologize for these errors.

