# Supplementary material for: PROTOCOL: Psychometric properties of instruments for measuring elder abuse and neglect in community and institutional settings: A systematic review
Source: Campbell Syst Rev. 2023 Jun 27;19(3):e1342. doi: 10.1002/cl2.1342 (PMC10296034; doi:10.1002/cl2.1342)
Supplement: Supplementary file 1 — Supporting information. [file CL2-19-e1342-s001.docx]

Appendices

## 1 Definition of elder abuse subtypes

Hall 2016

| Abuse subtype | Definition |
| --- | --- |
| Physical | Elder experiences illness, pain, injury, functional impairment, distress, or death due to the intentional use of physical force and includes acts such as hitting, kicking, pushing, slapping, and burning. |
| Emotional | Verbal or nonverbal behaviours that inflict anguish, mental pain, fear, or distress on an older adult. Examples include humiliation or disrespect, verbal and non-verbal threats, harassment, and geographic or interpersonal isolation. |
| Financial | Illegal, unauthorised, or improper use of an elder’s money, benefits, belongings, property, or assets for the benefit of someone other than the older adult. |
| Sexual | Forced or unwanted sexual interaction of any kind with an older adult. This action may include unwanted sexual contact, penetration, or non-contact acts such as sexual harassment. |
| Neglect | Failure to meet an older adult’s basic needs. These needs include food, water, shelter, clothing, hygiene, and essential medical care. |

## 2 COSMIN definitions of domains, measurement properties, and aspects of measurement properties

(Mokkink 2018)

| Domain | Measurement property | Aspect of a measurement  property | Definition |  |
| --- | --- | --- | --- | --- |
| Reliability |  |  | The degree to which the measurement is free from measurement error. |  |
|  | Internal consistency |  | The degree of the  interrelatedness among the items. |  |
|  | Reliability |  | The proportion of the total variance in the measurements is due to ‘true’† differences between patients. |  |
|  | Measurement error |  | The systematic and random error of a patient’s score that is not attributed to true changes in the construct is to be measured. |  |
| Validity |  |  | The degree to which a PROM measures the construct(s) it purports to measure. |  |
|  | Content validity |  | The degree to which the content of a PROM is an adequate reflection of the construct to be measured. |  |
|  |  | Face validity | The degree to which (the items of) a PROM look like an adequate reflection of the construct to be measured. |  |
|  | validity |  | The degree to which the scores of a PROM are consistent with hypotheses (for instance, concerning internal relationships, relationships to scores of other instruments, or differences  between relevant groups) based on the assumption that the PROM  validly measures the construct to be measured. |  |
|  |  | Structural  validity | The degree to which the scores of a PROM are an adequate reflection of the dimensionality of the construct to be measured. |  |
|  |  | Hypotheses  testing | Idem construct validity |  |
|  |  | Cross-cultural  validity | The degree to which the  performance of the items on a translated or culturally adapted PROM is an adequate reflection of the performance of the items of the original version of the PROM. |  |
|  | Criterion  validity |  | The degree to which the scores of a PROM are an adequate reflection of a ‘gold standard’. |  |
| Responsiveness |  |  | The ability of a PROM to detect change over time in the construct is to be measured. |  |
|  | Responsiveness |  | Idem responsiveness |  |
| Interpretability* |  |  | Interpretability is the degree to which one can assign qualitative meaning ‐ that is, clinical or commonly understood connotations – to a PROM’s quantitative scores or change in scores. |  |

The word ‘true’ must be seen in the context of the CTT, which states that any observation comprises two components – a true score and an error associated with the observation.

‘True’ is the average score obtained if the scale were given an infinite number of times. It refers only to the consistency of the score and not to its accuracy

* Interpretability is not considered a measurement property, but an important characteristic of a measurement instrument

## 3 PubMed search strategy

| PubMed | ((( (“old* person*“ OR “old* people*“ OR “old* individual*“ OR aged OR gerontol* OR elder* OR geriatric* OR ageing OR aging OR senior* OR retir* OR octogenarian* OR nonagenarian* OR septuagenarian* OR sexagenarian* OR “old* man*“ OR “old* woman*“ OR “old* men*“ OR “old* women*“ OR “old* minorit*“ OR senescent OR senile* OR “older adult*“ OR “later life*“ OR “later in life*“) N3 (neglect* OR abus* OR maltreat* OR restrain* OR mistreat* OR illtreat* OR “ill treat*“ OR ill-treat* OR scorn* OR hit* OR harm* OR damage* OR victimis* OR victimiz* OR trauma* OR manhandl* OR isolate* OR exclu* OR exploit* OR bully* OR violen* OR self-neglect* OR intimidat* OR threaten* OR distress* OR abandon* OR fraud* OR rape* OR scam* OR crime* OR cheat* OR extort* OR hoax OR swindle* OR coerce* OR crime* OR criminal* OR assault* OR “bodily harm” OR felony OR batter* OR attack* OR beat*))) OR (elder abuse[MeSH Terms]))  AND  (((psychometr*[Title/Abstract] OR “outcome assessment”[Title/Abstract] OR “observer variation”[Title/Abstract] OR reproducib*[Title/Abstract] OR reliab*[Title/Abstract] OR unreliab*[Title/Abstract] OR valid*[Title/Abstract] OR “coefficient of variation”[Title/Abstract] OR coefficient[Title/Abstract] OR homogeneity[Title/Abstract] OR homogeneous[Title/Abstract] OR “internal consistency”[Title/Abstract] OR “cronbach alpha”[Title/Abstract] OR “cronbach alphas”[Title/Abstract] OR “items correlation*“[Title/Abstract] OR “items selection*“[Title/Abstract] OR “item reduction*“[Title/Abstract] OR “test retest”[Title/Abstract] OR stability[Title/Abstract] OR interrater[Title/Abstract] OR inter-rater[Title/Abstract] OR intrarater[Title/Abstract] OR intra-rater[Title/Abstract] OR intertester[Title/Abstract] OR inter-tester[Title/Abstract] OR intratester[Title/Abstract] OR intra-tester[Title/Abstract] OR interobserver[Title/Abstract] OR inter-observer[Title/Abstract] OR intraobserver[Title/Abstract] OR intra-observer[Title/Abstract] OR interexaminer[Title/Abstract] OR inter-examiner[Title/Abstract] OR intraexaminer[Title/Abstract] OR intra-examiner[Title/Abstract] OR interindividual[Title/Abstract] OR inter-individual[Title/Abstract] OR intraindividual[Title/Abstract] OR intra-individual[Title/Abstract] OR interparticipant[Title/Abstract] OR inter-participant[Title/Abstract] OR intraparticipant[Title/Abstract] OR intra-participant[Title/Abstract] OR kappa[Title/Abstract] OR kappa’s[Title/Abstract] OR kappas[Title/Abstract] OR “repeated measure”[Title/Abstract] OR “repeated measures”[Title/Abstract] OR “repeated findings”[Title/Abstract] OR “repeated results”[Title/Abstract] OR “repeated test”[Title/Abstract] OR “repeated tests”[Title/Abstract] OR generaliza*[Title/Abstract] OR generalisa*[Title/Abstract] OR concordance[Title/Abstract] OR “intraclass-correlation”[Title/Abstract] OR discriminative[Title/Abstract] OR “known group”[Title/Abstract] OR “factor analysis”[Title/Abstract] OR “factor analyses”[Title/Abstract] OR “factor structure”[Title/Abstract] OR “factor structures”[Title/Abstract] OR dimension*[Title/Abstract] OR subscale*[Title/Abstract] OR “multitrait analysis”[Title/Abstract] OR “multitrait analyses”[Title/Abstract] OR “scaling analysis”[Title/Abstract] OR “scaling analyses”[Title/Abstract] OR “item discriminant”[Title/Abstract] OR “interscale correlation*“[Title/Abstract] OR error[Title/Abstract] OR errors[Title/Abstract] OR “individual variability”[Title/Abstract] OR “interval variability”[Title/Abstract] OR “rate variability”[Title/Abstract] OR “variability analysis”[Title/Abstract] OR “variability values”[Title/Abstract] OR “uncertainty measurement”[Title/Abstract] OR “standard error of measurement”[Title/Abstract] OR sensitiv*[Title/Abstract] OR responsive*[Title/Abstract] OR “limit detection”[Title/Abstract] OR “minimal detectable concentration”[Title/Abstract] OR interpretab*[Title/Abstract] OR “Minimal important”[Title/Abstract] OR “minimally important”[Title/Abstract] OR “clinical important”[Title/Abstract] OR “clinically important”[Title/Abstract] OR “Minimal significant”[Title/Abstract] OR “Minimally significant”[Title/Abstract] OR “clinical significant”[Title/Abstract] OR “clinically significant”[Title/Abstract] OR “minimal detectable”[Title/Abstract] OR “minimally detectable”[Title/Abstract] OR “clinical detectable”[Title/Abstract] OR “clinically detectable”[Title/Abstract] OR Change[Title/Abstract] OR difference[Title/Abstract] OR “meaningful change”[Title/Abstract] OR “ceiling effect”[Title/Abstract] OR “floor effect”[Title/Abstract] OR “Item response model”[Title/Abstract] OR IRT[Title/Abstract] OR Rasch[Title/Abstract] OR “Differential item functioning”[Title/Abstract] OR DIF[Title/Abstract] OR “computer adaptive testing”[Title/Abstract] OR “item bank”[Title/Abstract] OR “cross-cultural equivalence”[Title/Abstract] OR “screening tool”[Title/Abstract] OR “screening assessment”[Title/Abstract] OR assessment[Title/Abstract] OR “assessment tool”[Title/Abstract] OR screening[Title/Abstract] OR “appraisal tool”[Title/Abstract])) OR ((((((psychometrics[MeSH Terms]) OR (assessment, outcome health care[MeSH Terms])) OR (observer variation[MeSH Terms])) OR (health status indicator[MeSH Terms])) OR (reproducibility of results[MeSH Terms])) OR (discriminant analysis[MeSH Terms]))) |
| --- | --- |

## 4 Table 1: Characteristics of the included measuring instruments

| Measurement instruments* (a reference to the first article) | Study | Construct(s)* | Target  population | Mode of  administration  (e.g., self‐report,  interview‐based,  parent/proxy  report etc.) | Recall  period | (Sub)scale  (s) (number  of items) | Response  options | Range of  scores/scoring | Original  language | Available  translations |
| --- | --- | --- | --- | --- | --- | --- | --- | --- | --- | --- |
| CTS | Ref 1….. |  |  |  |  |  |  |  |  |  |
|  | Ref 2….. |  |  |  |  |  |  |  |  |  |

* Construct(s): Types and subtypes of abuse and measured by the instrument

## 5 Table 2: Characteristics of the included study populations

| Measurement instruments | Ref | Author, publication year | Study design | Population | | | Abuse characteristics | | Instruments administration | | | |
| --- | --- | --- | --- | --- | --- | --- | --- | --- | --- | --- | --- | --- |
|  |  |  |  | Sample size (N) | Gender % (female, male) | Victim/ perpetrator / 3rd person, i.e. staff | Types and subtypes of abuse and measured | Definitions | Study setting | Country | Language | Response rate |
| A | 1 |  |  |  |  |  |  |  |  |  |  |  |
|  | 2 |  |  |  |  |  |  |  |  |  |  |  |
| B | 1 |  |  |  |  |  |  |  |  |  |  |  |

## 6 Table 3: COSMIN Risk of Bias checklist

| **Content validity**  Box 1: PROM Development  Box 2: Content validity |
| --- |
| **Internal structure**  Box 3: Structural validity  Box 4: Internal consistency  Box 5: Cross-cultural validity/measurement invariance |
| **Remaining measurement properties**  Box 6: Reliability  Box 7: Measurement error  Box 8: Criterion validity  Box 9: Hypotheses testing for construct validity  Box 10. Responsiveness |

The definition of each box is shown in Appendix 2
